# Detection and Antimicrobial Susceptibility of Carbapenem-Resistant Organisms Isolated in the Center of Care and Protection of Orphan Children, Vietnam

**DOI:** 10.1155/ijm/3147068

**Published:** 2025-09-19

**Authors:** Nguyen Van Kim, Tran Dang Thang, Cao Thang Long, Katiya Ivanovitch, Stephen Baker, Pirom Noisumdaeng

**Affiliations:** ^1^Faculty of Public Health, Thammasat University, Pathum Thani, Thailand; ^2^Hung Vuong Hospital, Ho Chi Minh City, Vietnam; ^3^Department of Medicine, University of Cambridge, Cambridge, UK; ^4^Thammasat University Research Unit in Modern Microbiology and Public Health Genomics, Thammasat University, Pathum Thani, Thailand

**Keywords:** bacteria, carbapenems, carbapenem-resistant organisms (CROs), environment, healthcare setting, healthcare workers, infection prevention and control, orphan children

## Abstract

Carbapenems are critical for treating patients infected with multidrug-resistant bacteria; however, the use of carbapenems has also facilitated the selection and spreading of carbapenem-resistant organisms (CROs), occasionally reported in healthcare settings. The study examined the CRO prevalence among healthcare workers (HCWs), orphan children patients, and the environment in an orphanage healthcare facility in Vietnam. A cross-sectional study was performed by collecting rectal swabs in 20 HCWs and 67 orphan patients, as well as in 175 randomly selected environmental samples. Chromogenic CARBA agars, blood agars, and a BD Phoenix Automated Microbiology System were employed for bacterial isolation and for identification and testing of antimicrobial susceptibility. In a total of 262 samples, 36 CROs (i.e., six carbapenem-resistant Enterobacterales [CRE] and 30 non-CRE) were detected. The CRO prevalence of 30.0% (6/20), 16.4% (11/67), and 10.86% (19/175) was shown in HCWs, orphan patients, and the environment, respectively. Most CROs detected in HCWs were CREs (66.7%, 4/6). Non-CRE cases, mainly *Acinetobacter baumannii*, were detected in orphan patients and in the orphanage healthcare environment. Out of 36 CRO isolates, 97.2% (35/36), 11.1% (4/36), and 13.9% (5/36) were identified as resistant to ertapenem, imipenem, and meropenem, respectively. This study was the first to show evidence-based CRO colonization with an epidemiological study in an orphanage healthcare facility in Vietnam. The finding of this study suggested that control and prevention programs, active surveillance, and routine monitoring for CROs should be implemented in healthcare establishments.

## 1. Introduction

Infection caused by antimicrobial-resistant (AMR) bacteria is a major global public health challenge [1]. Methicillin-resistant *Staphylococcus aureus* (MRSA), vancomycin-resistant Enterococci (VRE), extended-spectrum beta-lactamase (ESBL)-producing Gram-negative bacteria, and carbapenem-resistant organisms (CROs) are frequently associated with community- and hospital-acquired infections (HAIs) [2–6]. Multidrug-resistant (MDR) bacteria are of primary concern as they harbor multiple mechanisms fostering resistance to several drug classes [7]. Carbapenem antimicrobials are critical for treating patients infected with MDR bacteria; however, carbapenem's use has also facilitated selection and spreading of CROs in healthcare settings. CROs are commonly associated with HAIs and substantial morbidity due to limited treatment options [8–11]. Specifically, carbapenem-resistant Enterobacterales (CRE) is a major problem in healthcare settings [2–5, 12]. The World Health Organization (WHO) has identified the ESBL-producing Enterobacterales and Gram-negative CROs (including CRE, carbapenem-resistant *Acinetobacter baumannii* [CRAB] and carbapenem-resistant *Pseudomonas aeruginosa* [CRPA]) as priority category pathogens [13, 14]. The presence of these bacteria is of concern for healthcare management because they influence the quality of care and cure and can trigger infection and colonization in healthcare settings [15]. Several previous studies demonstrated that CROs are frequently detected in patients and in healthcare environments [14–17].

The prevalence of carbapenem-resistant *Escherichia coli* and *Klebsiella pneumoniae* is increasing and is dependent on the region studied. Surveillance studies conducted in Europe reported that the prevalence of *K. pneumoniae* increased significantly from 0.2% to 33.4% between 2015 and 2017 [18]. At the same time, the prevalence increased from 1.3% to 28.6% in Latin America [19], while in North America, it went from 3.1% to 4.9% [20]. Notably, an increasing prevalence was reported of carbapenem-resistant *E. coli* of 0%–7% [18], 0.4%–9% [19], and 0.2%–0.4% [20] in Europe, Latin America, and North America, respectively. The prevalence of carbapenem-resistant *K. pneumoniae* in Asian countries, such as India, Nepal, Pakistan, and Vietnam, is currently estimated to range from 0% to 52% [21]. Similarly, the prevalence of carbapenem-resistant *E. coli* in Asian countries, including Vietnam, has been reported in the range of 0%–34% [21]. However, those reports were mostly obtained from patients through the surveillance systems because data are limited in Vietnam about carbapenem-resistant bacteria in healthcare workers (HCWs) and healthcare environments.

This study investigated the prevalence of CROs among HCWs, orphan patients, and the healthcare environment at the Vietnam Center of Care and Protection for orphan children. This center is a healthcare setting for otherwise abled and immunosuppressed children with cerebral palsy. These patients often have episodes of recurrent pneumonia requiring long-term antimicrobial treatment and prolonged hospital stays. The child patients lived and interacted (while eating and playing) at the center for long periods. In some cases, the child patients needed assistance from HCWs to consume their food. As a result, the CRO transmission may have also occurred because HCWs and patients failed to comply with hygiene regulation. It is, therefore, of utmost importance to prevent CRO by enforcing hygiene measures among HCWs, patients, and the general healthcare environment.

## 2. Materials and Methods

### 2.1. Ethical Approval

This study received an approval from the Board of Directors on Ethics in Biomedical Research at Thien Phuoc Nhan Ai Center of Care and Protection of Disabled Children, Vietnam, under the approval number 026/2565. Written informed consent was obtained from HCWs and the children's guardians.

### 2.2. Study Design, Human Subjects, and Environmental Samples

A cross-sectional study was performed to investigate the CRO prevalence among HCWs, patients, and environment. At the Vietnam's Thien Phuoc Nhan Ai Center of Care and Protection of Disabled Children, there are 20 HCWs and 67 pediatric patients. Due to the small and limited population size, all eligible individuals were invited to participate in the study, with no prior sample size calculation was conducted. For environmental samples, we initially surveyed, observed, and determined the area of highly potential contaminated area, which identified of 307 environmental samples in the center as in Table S1. After that, we calculated the sample size based on the Yamane formula, which is *n* = *N*/1 + *N*.*e*^2^, where *n* is the sample size required for the study, depending on *N*. *N* is the total number of environmental samples observed and determined in our study site, the Center of Care and Protection of Orphan Children, and *e* = 0.05, allowable error (%). Hence, *N* was 307; therefore, the sample sizes of the randomly environmental samples required for our study was 175 (*n*). The samples were collected between September and December 2022 at the site of the healthcare center for orphan children with mental and physical disorders.

For specimen collection from HCWs and patients, rectal specimens were obtained from participants by using sterile rectal cotton sampling swabs. The sampling swab was inserted approximately 1 cm into the anal canal and then rotated slowly for 10 s. For environmental samples, an area of approximately 10 cm^2^ from surfaces presumptive of high contamination was swabbed for each environmental sample. All swab specimens were preserved in Amies transport medium and stored chilled in a container before transfer to the Microbiology Laboratory Unit at Hung Vuong Hospital within 6 h of the collection.

### 2.3. Microbiological Testing

Microbiological testing was performed at the Microbiology Laboratory Unit, Hung Vuong Hospital in Ho Chi Minh City. MELAB Chromogenic CARBA agar plates (MELAB Diagnostics, Lavicom Company, Ltd.), blood agar plates (Nam Khoa Biotek. Ltd.), and the BD Phoenix Automated Microbiology System (Becton Dickinson [BD], United States) were used to screen, enrich, and confirm the presence of carbapenem-resistant bacteria. Briefly, rectal and environmental swabs were inoculated on MELAB Chromogenic CARBA plates and incubated overnight at 37°C. Indicative colonies were picked up and cultured onto blood agar. Bacterial species identification and phenotypic antimicrobial susceptibility testing were conducted by using the NMIC-500 panel of the BD Phoenix Automated Microbiology System according to the manufacturer's instructions [22, 23]. For the detection of beta-lactamase-producing organisms, the BD Phoenix detection test uses the principles of Ambler-class specific beta-lactamase inhibition and Ambler-class specific antibiotic resistance to detect the presence of a carbapenemase and to derive the Ambler class of the carbapenemase. The test is a qualitative confirmatory growth-based test intended to phenotypically detect carbapenemase enzyme expression in Enterobacteriaceae, *P. aeruginosa*, and *A. baumannii*. The test is intended to determine whether an organism is positive or negative for carbapenemase production and, when positive, provide the Ambler classification (i.e., Class A, Class B, or Class D) (https://ecp-eg.com/wp-content/uploads/2020/09/manual-in-english.pdf). Bacterial reference strains, *E. coli* ATCC 25922, *K. pneumoniae* ATCC BAA-1705, and *P. aeruginosa* ATCC 27853, were used as bacterial standard controls.

### 2.4. Data Analysis

Descriptive statistics were used to examine the CRO prevalence among HCWs, children patients, and the healthcare environment. Result interpretation on antimicrobial susceptibility in the BD Phoenix Automated Microbiology System was based on minimum inhibitory concentration (MIC) measurement, which was analyzed by using Epicenter data management software version 7.22A (BD Diagnostic Systems) [22, 23]. The interpretation values of panel MIC data were established by the Clinical and Laboratory Standard Institute (CLSI) as follows: susceptible (S), intermediate (I), or resistant (R) result classification, while the result of “X” means that the MIC value is outside the breakpoints for the selected standard and cannot be interpreted as SIR results [23].

## 3. Results

### 3.1. Detection of CROs in HCWs, Patients, and the Environment

Of the total 262 samples, 67 rectal swabs (20 HCWs and 67 patients) and 175 environmental samples, 36 CROs (i.e., six strains of CREs and 30 strains of non-CREs) were isolated. Among 87 rectal swab samples, the CRO detection rates were 30.0% (6/20) and 16.4% (11/67) in HCWs and children patients, respectively (Table 1). In HCWs, four CREs, including three isolates of *E. coli* (15%, 3/20) and one *Enterobacter cloacae* (5%, 1/20) were detected. Two isolates of *A. baumannii* (10%, 2/20) were also identified in the HCWs. In orphan children patients, 11 CRO isolates, that is, *E. coli*, *A. baumannii*, *P. aeruginosa*, *Burkholderia cepacia* complex, and *K. pneumoniae* were isolated, resulting in a prevalence of 1.49% (1/67), 10.45% (7/67), 1.49% (1/67), 1.49% (1/67), and 1.49% (1/67), respectively. In addition, 19 CROs (10.9%, 19/175) including *A. baumannii* (4.57%), *Alcaligenes faecalis* (2.29%), *P. aeruginosa* (0.57%), *P. putida* (2.29%), and *Stenotrophomonas maltophilia* (1.14%) were identified in the environmental samples (Table 1).

### 3.2. Detection and Distribution of CROs in Environmental Samples

The detection rate of CROs in the environment of the Center of Care and Protection of Orphan Children was 10.86% (19/175). Most isolates detected were *A. baumannii* (4.57%, 8/175), followed by *A. faecalis*, *P. putida*, *S. maltophilia*, and *P. aeruginosa*. Specifically, we detected *A. baumannii* on the faucet handles and the upper part of the washing machine and on the first floor of the center. We also found them in the hands of HCWs and children, children's beds, and the dining table. *A. faecalis* was from outside of the feeding tube and the inner wall of the piston food pump, on the toilet seats, and a child table for lunch and dinner. Similarly, in the general ward, *P. putida* was isolated from a toy, chair, dining table, and a soil sample inside a small chicken farm located in the center. *P. aeruginosa* was isolated from the faucet handle for cooking and cleaning vegetables in the kitchen and on the general floor. Finally, on the general floor, *S. maltophilia* was detected on a chair in the kitchen and on the toothbrush shelf for children (Table 2).

### 3.3. Antimicrobial Susceptibility Test

We analyzed the phenotypic antimicrobial susceptibility among 36 bacterial isolates against 25 antimicrobials, i.e., ertapenem (ETP), imipenem (IP), meropenem (MP), amikacin (AK), ampicillin (AM), amoxicillin–clavulanic acid (AMC), aztreonam (AZM), cefazolin (CZ), cefepime (FEP), cefoxitin (FOX), ceftazidime (CAZ), ceftazidime/avibactam (CZA), ceftriaxone (CRO), cefuroxime (CXM), ciprofloxacin (CIP), colistin (CST), fosfomycin (FO), gentamicin (GEN), levofloxacin (LVX), minocycline (MIN), nitrofurantoin (NFN), norfloxacin (NOR), piperacillin/tazobactam (TZP), tigecycline (TGC), and trimethoprim–sulfamethoxazole (SXT) using a BD Phoenix Automated Microbiology System. MDR was evaluated according to the MDR definition in which bacteria resist one or more agents in three or more antimicrobial classes. Overall, the AMR profiles demonstrated MDR bacteria detected in the study. All isolates (100%) of *E. coli*, *K. pneumoniae*, *E. cloacae*, *P. putida*, *P. aeruginosa*, *B. cepacia* complex, and *S. maltophila* were resistant to AM and AMC; and they were resistant to at least one of the tested drugs in first- to fourth-generation cephalosporins, aminoglycosides, and fluoroquinolones (Table 3). All isolates (100%) of *A. baumannii* and *A. faecalis* were resistant to ETP, AM, and AZ, and most isolates showed varying degrees of AMR patterns (Table 3).

Focusing on the CROs, of the 36 isolates, 97.2% (35/36), 11.1% (4/36), and 13.9% (5/36) were identified as resistant to ETP, IP, and MP, respectively. We detected *E. coli* class D in one child and one HCW and *E. coli* class B in one HCW. Three *E. coli* isolates (75%, 3/4) exhibited resistance to ETP and intermediate resistance to IM. All isolates of *K. pneumoniae* and *E. cloacae* were resistant to all carbapenems. In addition, all isolates of *A. baumannii*, *B. cepacia* complex, *A. faecalis*, *P. putida*, *P. aeruginosa*, and *S. maltophila* were resistant to ETP. The antimicrobial susceptibility profiles are described in Table 4.

## 4. Discussion

The emergence and ongoing spread of CROs is a major challenge in healthcare management. CROs are frequently identified in patients and the healthcare environment [24, 25]. Importantly, the community-acquired infections and HAIs caused by CROs/CRE have been previously reported and frequently observed in the developing countries where unregulated antibiotic use occurs [26–28]. We, therefore, hypothesized that the colonization, spreading, and intertransmission between patients, HCWs, and the environment possibly occurred. As a result, the study investigated the prevalence of CROs in Vietnam's healthcare setting where carbapenems were used to treat critically ill patients.

Our study reported that among 87 human rectal swab samples, the prevalence of CROs was 30.0% and 16.4% in HCWs and patients, respectively. The result was comparable to a previous study which showed evidence of 25% antibiotic-resistant Gram-negative bacteria, mainly ESBL/AmpC *β*-lactamase-producing *E. coli*, in HCWs working in an Intensive Care Unit in Southern Vietnam [29]. It implied that HCWs might serve as a carriage of AMR bacterial colonization and transmission. In contrast, a low prevalence and no HCW carrier of CROs was previously reported in developed countries, suggesting that the risk of CRO colonization and transmission in HCWs is limited [30, 31]. In patients, the prevalence of CROs was 14.92%, mostly identified as *A. baumannii*, which has been usually found in the healthcare setting, especially ICUs in Vietnam [32, 33]. The CROs, including CRE, were also frequently found in hospital-based pediatric wards. The prevalence survey implemented for six pediatric ICUs of three Vietnamese pediatric hospitals reported that there were 454 HAI cases, and 276 bacterial strains were isolated from those pediatric cases. Most bacterial species were *K. pneumoniae*, *Pseudomonas* spp., *Acinetobacter* spp., and *S. aureus*. The study detected carbapenem resistance in 55% of *K. pneumoniae*, 71% of *P. aeruginosa*, and 65% of *A. baumannii* isolates [32, 33]. Compared to our study, the results suggested that the detection rate of CROs might be significant, depending on the sources of specimen collection (hospital ICU-based setting vs. healthcare setting). Significantly, CROs detected in patients were mostly non-CREs, which were typically present in the environment, suggesting a possible transmission of CROs between patients and the healthcare environment.

This study found the CRO prevalence of 10.86% in the environment of the healthcare setting. The majority of isolates detected were *A. baumannii*. One survey in a Ghanaian Tertiary Hospital showed the high prevalence of *A. baumannii* carrying *bla*_NDM-1_ in different wards at many sites, including desk surfaces, tap handle, and bed handles [30], and *A. baumannii* appeared in the environmental sites of healthcare settings such as suctioning equipment, bedside tables, and shower trolleys [34]. Other studies also found carbapenem-resistant *A. baumannii* and *P. aeruginosa* in drains, sinks, and faucets, which act as main sources for transmitting CROs from the environment to HCWs or patients [25, 35, 36]. These study results showed that environmental *A. baumannii* isolates were possibly spread to patients or HCWs in healthcare settings through healthcare activities [37]. Our findings demonstrated that CROs, especially *A. baumannii*, were present in the environment, patients, and HCWs. We also could isolate *A. baumannii* on HCWs and patient's hands. This result implied that the carbapenem-resistant *A. baumannii* in our study could be a transmission source from the environment to HCWs and patients or conversely. However, the genetic analysis of the individual clones should be conducted to confirm the transmission among HCWs, patients, and the environment. Moreover, we detected the persistence of environmental CROs, including opportunistic pathogens *A. faecalis*, *P. aeruginosa*, and *P. putida*. A previous study showed that these were frequently found in healthcare settings, including soil or hospital environments such as respirators, medical equipment, hemodialysis systems, and intravenous solutions [38–40]. Therefore, cleaning faucets, chairs, and equipment used for caring, preparing food, and disinfecting medical equipment used for patients are the necessary procedures to prevent the spread of these bacteria [41].

In addition to CRO detection, our study demonstrated phenotypic AMR profiles among those bacterial isolates to various classes of antibiotics. Overall, the MDR bacteria were observed to show high resistance rates against first- to fourth-generation cephalosporins (*β*-lactam antimicrobials), aminoglycosides, and fluoroquinolones. Our study found class B metallo- and class D *β*-lactamases producing *E. coli* isolated from participants. They showed multi-resistance to *β*-lactam antimicrobials, implying that they might produce ESBL and/or carbapenemase enzymes such as IMP, VIM, NDM, and OXA [42]. All isolates of *K. pneumoniae*, *E. cloacae*, *P. putida*, *P. aeruginosa*, *B. cepacia* complex, and *S. maltophila*, and most of *A. baumannii* human isolates were resistant to at least one of the tested drugs in three classes. The results corresponded to previous studies demonstrating high resistance rates (79.9%–100%) of Gram-negative bacteria such as *E. coli*, *P. aeruginosa*, and *A. baumannii* isolates to multiple classes of antibiotics [43]. Interestingly, the phenotypic AMR profiles among *P. aeruginosa* and *A. baumannii* isolates from HCW, patients, and the environment demonstrated similar patterns, implying that the transmission of patient–HCW–environment might play an important role in spreading AMR in healthcare settings.

However, this study has several limitations. First, it was conducted at a single study site with a limited human sample size, which may affect the generalizability of the findings, including the observed prevalence of CRO and the diversity of bacterial species identified. Additionally, potential biases related to the selection of environmental sampling locations and timing may have influenced the accuracy and representativeness of bacterial identification. Further genotypic characterization of bacterial CRO strains using whole-genome sequencing (WGS) is warranted to investigate the presence of clinically significant antibiotic resistance genes.

## 5. Conclusions

This is the first report showing the CRO colonization in HCWs, patients, and the healthcare environment of the orphanage healthcare center in Vietnam. Various Gram-negative bacterial species were also identified. Most of those detected bacteria were opportunistic pathogens that can potentially cause infections in immunocompromised persons. It is suggested that the control and prevention of CRO colonization should be implemented to reduce CRO spread among HCWs, children patients, and the healthcare environment. The hand hygiene compliance for HCWs and patients, and environmental cleaning should be mandatory to prevent and control the CRO colonization and transmission. All HCWs should be trained and practiced to follow infection control guidelines. In addition, to decrease the environmental CRO contamination, the cleaners should be trained to perform basic hygiene tasks to effectively decontaminate items used for patient care. Furthermore, to proactively prevent and control CRO colonization, there should be routine surveillance and monitoring of HCWs, patients, and the healthcare environment setting. The molecular aspects, such as genotyping by WGS, should also be applied to investigate the potential clonal spreading and transmissions of CROs among patient-HCWs-environment.

## Figures and Tables

**Table 1:** Detection of CROs in HCWs, orphan children patients, and environmental samples in the Center of Care and Protection of Orphan Children, Vietnam.

**Bacterial species**	**Number of isolates detected**	**Frequency of detecting CROs in**		
		**HCWs (** **N** = 20**) (%)**	**Orphan children patients (** **N** = 67**) (%)**	**Environment (** **N** = 175**) (%)**
*E. coli*	4	3 (15%)	1 (1.49%)	—
*E. cloacae*	1	1 (5%)	—	—
*A. baumannii*	17	2 (10%)	7 (10.45%)	8 (4.57%)
*A. faecalis*	4	—	—	4 (2.29%)
*P. aeruginosa*	2	—	1 (1.49%)	1 (0.57%)
*P. putida*	4	—	—	4 (2.29%)
*S. maltophilia*	2	—	—	2 (1.14%)
*B. cepacia* complex	1	—	1 (1.49%)	—
*K. pneumoniae*	1	—	1 (1.49%)	—

Abbreviations: *A. baumannii*, *Acinetobacter baumannii*; *A. faecalis*, *Alcaligenes faecalis*; *B. cepacia*, *Burkholderia cepacia* complex; CRO, carbapenem-resistant organism; *E. cloacea*, *Enterobacter cloacea*; *E. coli*, *Escherichia coli*; *K. pneumoniae*, *Klebsiella pneumoniae*; *P. aeruginosa*, *Pseudomonas aeruginosa*; *P. putida*, *Pseudomonas putida*; *S. maltophilia*, *Stenotrophomonas maltophilia*.

**Table 2:** Detection and distribution of CROs in healthcare environmental samples.

**Environmental sampling**	**Number of samples**	**CRO**	**Number of isolates detected**	**% frequency of detection**
Faucet handle for hand washing	13	*A. baumannii*	1	15.4 (2/13)
Faucet handle for cooking and cleaning vegetable in kitchen		*P. aeruginosa*	1	
Electric train game for children	8	*P. putida*	1	12.5 (1/8)
Washing machine	2	*A. baumannii*	1	50 (1/2)
Children Chair 1 for lunch and dinner in kitchen	24	*P. putida*	1	8.3 (2/24)
Children Chair 2 for lunch and dinner in kitchen		*S. maltophilia*	1	
Soil sample collected inside chicken small farm	1	*P. putida*	1	100 (1/1)
Contact area between hand of HCW and hand of children	7	*A. baumannii*	1	28.6 (2/7)
Contact area in palm of hand of HCWs		*A. baumannii*	1	
Sample collected from outpart of feeding tube and inner wall of piston used to pump food to feed a disabled child	1	*A. faecalis*	1	100 (1/1)
Toilet bowl in Room 2	8	*A. faecalis*	1	25 (2/8)
Toilet bowl in Room 3		*A. faecalis*	1	
Toothbrush shelf for children in Room 1	4	*S. maltophilia*	1	100 (4/4)
Children wood bed in Room 4	35	*A. baumannii*	1	8.6 (3/35)
Children plastic Bed 1 in Room 4		*A. baumannii*	1	
Children plastic Bed 2 in room 4		*A. baumannii*	1	
Tables for lunch and dinner	12	*A. baumannii*	1	25 (3/12)
		*A. faecalis*	1	
		*P. putida*	1	
Wall of all rooms	16	Not detected	0	0 (0/16)
Floor	5	Not detected	0	0 (0/5)
Door handle of rooms and toilets	8	Not detected	0	0 (0/8)
Pillow	31	Not detected	0	0 (0/31)

*Note:* Summary: Environmental samples (*N* = 175), 19 (10.9%, 19/175) CROs detected.

**Table 3:** Antimicrobial susceptibility profiles of CROs isolated in the study.

**Bacterial species (strain code)**	**Isolation source**	**Antimicrobial susceptibility pattern**																								
		**ETP**	**IP**	**MP**	**AK**	**AM**	**AMC**	**AZM**	**CZ**	**FEP**	**FOX**	**CAZ**	**CZA**	**CRO**	**CXM**	**CIP**	**CST**	**FO**	**GEN**	**LVX**	**MIN**	**NFN**	**NOR**	**TZP**	**TGC**	**SXT**
*E. coli* class D (C4 003)	Patients	R	I	S	S	R	R	R	R	S	R	R	S	R	R	I	X	S	S	X	S	S	S	R	S	R
*E. coli* class D (H 006)	HCWs	R	S	S	S	R	R	R	R	S	R	R	S	R	R	R	X	S	R	R	S	S	R	R	S	R
*E. coli* class B (H 019)	HCWs	S	I	S	S	R	R	S	X	S	S	S	S	S	S	R	X	S	S	R	S	S	S	I	S	S
*E. coli* (H 007)	HCWs	R	I	S	S	R	R	R	R	R	R	R	S	R	R	R	X	S	R	R	S	S	R	R	S	R
*P. aeruginosa* (C1 003)	Patients	R	I	S	S	R	R	S	R	S	R	S	S	R	R	S	X		S	S	R	R	S	S		R
*P. aeruginosa* (330)	Environment	R	S	S	S	R	R	R	R	S	R	S	S	R	R	S	X		S	I	R	R	S	S	R	R
*A. baumannii* (C1 007)	Patients	R	S	R	S	R	R	R	R	I	R	I		X	R	S	X	R	S	S	S	R		I		S
*A. baumannii* (C1 013)	Patients	R	S	I	S	R	S	R	R	R	R	R		X	R	S	R	R	S	S	S	R		S		S
*A. baumannii* (C2 012)	Patients	R	S	S	S	R	S	R	R	S	R	S		X	R	S	X	R	S	S	S	R		S		S
*A. baumannii* (C3 002)	Patients	R	S	S	S	R	S	R	R	S	R	S		X	R	S	X	R	S	S	S	R		S		S
*A. baumannii* (C2 020)	Patients	R	S	S	S	R	S		R	I		S		X		S	X		S		S			S		S
*A. baumannii* (C3 022)	Patients	R	S	S	S	R	S		R			S		X		S	X	R	S		S					S
*A. baumannii* (C3 011)	Patients	R	S	S	S	R	S		R			S		X		S	X		S		S			S		S
*A. baumannii* (H 013)	HCWs	R	S	S	S	R	S		R			S		X		S	X	R	S		S					S
*A. baumannii* (H 009)	HCWs	R	S	S	S	R	S		R	S		R		X		S	X		S		S			S		S
*A. baumannii* (292)	Environment	R	S	S	S	R	S	R	R	S	R	I		X	R	S	X	R	S	S	S	R		S		S
*A. baumannii* (392)	Environment	R	S	S	S	R	R		R	I		S		X		S	X		S		S			I		S
*A. baumannii* (373)	Environment	R	S	S	S	R	S		R	S		S		X		S	X		S		S			S		S
*A. baumannii* (379)	Environment	R	S	S	S	R	S		R	S		S		X		S	X		S		S			I		S
*A. baumannii* (381)	Environment	R	S	S	S	R	S		R	S		S		X		S	X		S		S			I		S
*A. baumannii* (315)	Environment	R	S	S	S	R	S	R	R	S	R	S		X	R	S	X	R	S	S	S	R		S		S
*A. baumannii* (403)	Environment	R	S	S	S	R	S		R			S		X		S	X	R	S		S					S
*A. baumannii* (419)	Environment	R	S	S	S	R	S		R	S		R		X		S	X		S		S			S		S
*B. cepacia* (C3 001)	Patients	R	S	S		R	R		R		R	S			R		R	R		S	S	R		R		S
*K. pneumoniae* (C4 003)	Patients	R	R	R	S	R	R	S	R	R	R	S	S	R	R	R	X		S	R	R	R	R	R	I	R
*E. cloacae* (H 008)	HCWs	R	R	R	S	R	R	R	R	R	R	R	R	R	R	R	X		R	X	S	R	S	R	S	R
*A. faecalis*(319)	Environment	R	S		S	R		R	R	R	R	R		X	R	S	X		S	S	S	R	S	I		S
*A. faecalis*(320)	Environment	R	S		S	R		R	R	R	R	R		X	R	S	X		S	S	S	R	S	I		S
*A. faecalis*(317)	Environment	R	S		S	R		R	R	R	R	R		X	R	S	X		S	S	S	R	S	I		S
*A. faecalis*(423)	Environment	R	S		S	R			R	S		R		X		S	X		S		S			S		S
*P. putida* (370)	Environment	R	S	S	S	R	R		R	S		S		X		S	X		S		S			S		R
*P. putida* (366)	Environment	R	S	S	S	R	R		R	S		S		X		S	X		S		S			S		R
*P. putida* (420)	Environment	R	S	S	S	R	R		R	S		S		X		S	X		S		S			S		R
*S. maltophilia* (107)	Environment	R	R	R	R	R	R	R	R		R	S		R	R			R	R	S	S	R		R		S
*S. maltophilia* (364)	Environment	R	R	R	R	R	R		R		R	S		R	R			R	R		S			R		S

*Note:* “Blank area” is an antibiotic that is not recommended for the treatment of infections. X means the MICs of the antibiotic concentration on the panel are not enough to answer the SIR result, which is usually outside the CLSI breakpoint.

Abbreviations: AK, amikacin; AM, ampicillin; AMC, amoxicillin–clavulanic acid; AZM, aztreonam; CAZ, ceftazidime; CIP, ciprofloxacin; CRO, ceftriaxone; CST, colistin;; CXM, cefuroxime; CZ, cefazolin; CZA, ceftazidime/avibactam; ETP, ertapenem; FEP, cefepime; FO, fosfomycin; FOX, cefoxitin; GEN, gentamicin; I, intermediate; IP, imipenem; LVX, levofloxacin; MIN, minocycline; MP, meropenem; NFN, nitrofurantoin; NOR, norfloxacin; R, resistance; S, susceptible; SXT, trimethoprim–sulfamethoxazole; TGC, tigecycline; TZP, piperacillin/tazobactam.

**Table 4:** Carbapenem susceptibility profiles of CROs detected in HCWs, patients, and the environment in the Center of Orphan Children, Vietnam.

**Bacterial species (number of isolate)**	**% resistance to carbapenems**		
	**ETP**	**IP**	**MP**
*E. coli* (*n* = 4)	75%	0%	0%
*P. aeruginosa* (*n* = 2)	100%	0%	0%
*A. baumannii* (*n* = 17)	100%	0%	5.88%
*B. cepacia* complex (*n* = 1)	100%	0%	0%
*K. pneumoniae* (*n* = 1)	100%	100%	100%
*E. cloacae* (*n* = 1)	100%	100%	100%
*A. faecalis* (*n* = 4)	100%	0%	0%
*P. putida* (*n* = 3)	100%	0%	0%
*S. maltophilia* (*n* = 2)	100%	100%	100%

Abbreviations: ETP, ertapenem; IP, imipenem; MP, meropenem.

## Data Availability

The data used to support the findings of this study are included within the article and supporting information.

## Supporting Information

Supporting Information Additional supporting information can be found online in the Supporting Information section. Table S1: Distribution of environmental samples and numbers of randomly collected environmental samples.
