# Supplementary material for: Detection and Antimicrobial Susceptibility of Carbapenem-Resistant Organisms Isolated in the Center of Care and Protection of Orphan Children, Vietnam
Source: Int J Microbiol. 2025 Sep 19;2025:3147068. doi: 10.1155/ijm/3147068 (PMC12474010; doi:10.1155/ijm/3147068)
Supplement: Supporting Information — Additional supporting information can be found online in the Supporting Information section. Table S1: Distribution of environmental samples and numbers of randomly collected environmental samples. [file 3147068.f1.docx]

**Supplementary Table S1.** Distribution of environmental samples, and numbers of randomly collected environmental samples

| **Type of environment observed** | **Total of determined environmental samples** | **Number of randomly collected samples** |
| --- | --- | --- |
| Children chairs for lunch and dinner in kitchen | 42 | 24 |
|  |  |  |
| Children wood and plastic bed in room 4 | 61 | 35 |
| Contact area (palm) between palm of HCW and children | 12 | 7 |
|  |  |  |
| Electric train game for children | 14 | 8 |
| Faucet handle (Fh) for washing hand & cooking and cleaning vegetable in kitchen | 23 | 13 |
|  |  |  |
| Sample collected from outpart of feeding tube and inner wall of piston used to pump food to feed a disabled child | 2 | 1 |
| Soil sample collected inside small chicken farm | 2 | 1 |
| Tables for lunch and dinner | 21 | 12 |
| Toilet bowl in room 2 and 3 | 14 | 8 |
| Toothbrush shelf for children in room 1 | 7 | 4 |
| Washing machine | 4 | 2 |
| Door handle of rooms and toilets | 14 | 8 |
| Floor | 9 | 5 |
| Pillow | 54 | 31 |
| Wall of all rooms | 28 | 16 |
| **Total** | 307 | 175 |
